# Enhancing Gait Symmetry via Intact Limb Kinematic Mapping Control of a Hip Disarticulation Prosthesis

**DOI:** 10.3390/biomimetics10100714

**Published:** 2025-10-21

**Authors:** Shengli Luo, Xiaolong Shu, Jiahao Du, Hui Li, Hongliu Yu

**Affiliations:** 1 School of Health Science and Engineering, University of Shanghai for Science and Technology, Shanghai 200093, China; lsl24835@usst.edu.cn (S.L.);; 2 Shanghai Engineering Research Center of Assistive Devices, Shanghai 200093, China

**Keywords:** hip disarticulation prosthesis, powered prosthesis, mapping control method, gait cycle, gait symmetry

## Abstract

Conventional hip disarticulation prostheses often require amputees to produce limited leg-lifting torque through exaggerated pelvic motion, resulting in complex control and pronounced gait abnormalities. To overcome the limitations, we present a mapping control strategy for a powered hip disarticulation prosthesis aimed at improving gait symmetry. A quaternion-based method was implemented to capture hip joint kinematics, while a gated recurrent unit (GRU) neural network was trained to model the kinematic relationship between the intact and prosthetic limbs, enabling biomimetic trajectory control. Validation experiments showed that trajectory similarity between predicted and actual motions increased with walking speed, reaching 98.12% at 3.0 km/h. Comparative walking tests revealed an 84.00% improvement in hip flexion angle with the powered prosthesis over conventional designs. Notable improvements in gait symmetry were observed: stride symmetry (measured by SI and RII) improved by 23.21% and 19.28%, respectively, while hip trajectory symmetry increased by 68.07% (SI) and 47.59% (RII). These results confirm that the GRU-based kinematic mapping model offers robust trajectory prediction and that the powered prosthesis significantly enhances gait symmetry, delivering more natural and biomimetic motion.

## 1. Introduction

Hip disarticulation prostheses are designed for high-level lower-limb amputees, including those with hip disarticulation, hemipelvectomy, or proximal femoral amputations [1,2]. Despite these clinical needs, current prosthetic solutions fail to restore natural gait patterns, leading to long-term compensatory movements and potentially causing secondary health complications [3,4]. Conventional designs are notoriously difficult to control and lack active torque generation during the swing phase. As a result, amputees must rely on exaggerated pelvic motion to lift the prosthetic limb, contributing to pronounced gait abnormalities [5,6]. A powered prosthetic hip joint capable of delivering active torque during swing offers strong potential to enhance gait symmetry and simplify control for users.

Research on intelligent hip disarticulation prostheses remains limited, partly due to the rarity of such amputations. Most existing devices are passive [7,8], lacking both agility and biomimetic function. Fully powered designs [9], while providing motorized hip and knee actuation, often exceed 11 kg in weight, posing a considerable burden on users. A microprocessor-controlled integrated hip-knee system [10] attempts to resolve torque deficits but suffers from structural limitations. Its four-bar linkage cannot replicate anatomical alignment, and its cable-driven system compromises durability. To address these shortcomings, this study proposes a biomimetic powered prosthetic hip joint that restores anatomical symmetry and improves gait function in hip disarticulation amputees. Table 1 presents a performance comparison of conventional hip disarticulation prostheses developed to date.

Early control strategies predominantly relied on finite-state machines using mechanical sensors, which, while reliable for rhythmic gait, often demonstrated limited adaptability to changing terrains or user intent. Control of intelligent lower-limb prostheses typically relies on physiological or mechanical signals [11,12,13]. While physiological signals have been effectively applied in knee prosthesis control [14,15], no established method exists for their use in hip disarticulation prostheses. Extremely short residual limb lengths in hip disarticulation amputees result in a limited number of available muscle groups. Mechanical signals, known for their stability and affordability, are widely used [12,16]; however, they introduce latency issues [17]. In hip disarticulation amputees, where residual limbs are extremely short and musculature is incomplete, this latency is further amplified due to limited motion monitoring capability. Conventional control strategies are thus inadequate. Mapping control offers a promising alternative by transmitting motion intent in real time via cross-limb coordination [18]. The study of human dynamic walking shows that the balance during locomotion results from complex bodily coordination [19], orchestrated by the central nervous system to regulate the cyclic transition between stance and swing phases [20]. This synchronicity makes intact-prosthetic limb mapping both feasible and biologically aligned. By capturing gait phase and joint trajectory features from the intact limb, mapping control enables real-time, coordinated prosthetic motion—making it particularly well-suited for hip disarticulation scenarios.

In summary, this study aims to address key limitations of conventional hip disarticulation prostheses, through three main objectives: (1) designing a biomimetic prosthetic hip joint to restore anatomical symmetry in bilateral lower limbs; (2) developing a kinematic mapping control framework based on quaternion-derived joint angles, using intact-limb motion to drive prosthetic movement; and (3) conducting walking experiments to evaluate the prosthesis’s impact on gait symmetry using standardized metrics.

## 2. Materials and Methods

### 2.1. Bionic Power Hip Disarticulation Prosthesis

**Figure 1:**
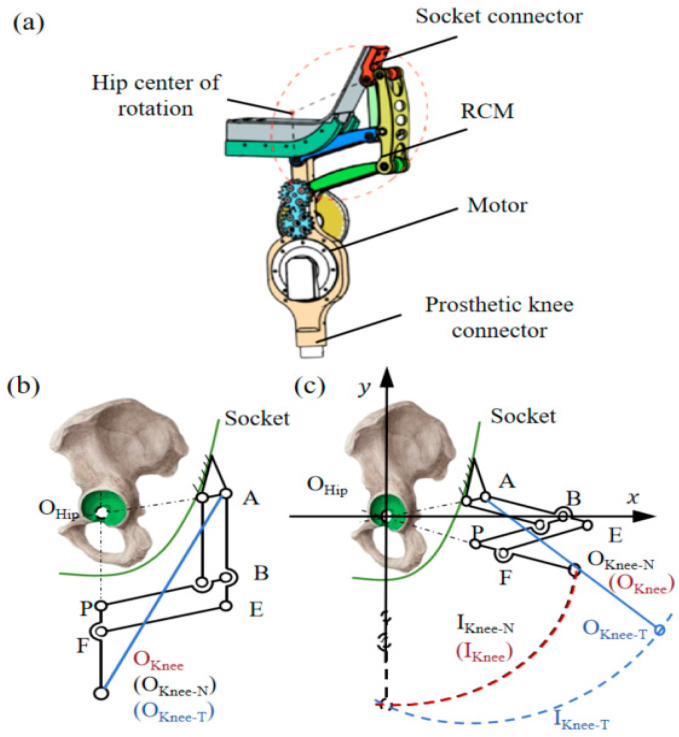
Structure and design principles of the biomimetic powered prosthetic hip joint: (**a**) Overall configuration of the powered biomimetic prosthetic hip joint. (**b**) Design comparison during the stance phase [6]: Conventional prosthetic knee (O_Knee-T_), natural human knee (O_Knee_), and the novel prosthetic knee (O_Knee-N_) positions align. (**c**) Design comparison during the swing phase [6]: Conventional prosthetic knee (O_Knee-T_) deviates from the natural knee (O_Knee_), while the novel prosthetic knee (O_Knee-N_) aligns with O_Knee_.

The biomimetic powered hip disarticulation prosthesis consists of a drive motor, a dual-parallelogram remote center of motion (RCM) mechanism [21], a socket connector, and a prosthetic knee connector, as shown in Figure 1. The drive motor actively generates torque during the swing phase, while the RCM mechanism restores anatomical alignment between the residual limb and the prosthetic structure [22]. The socket connector provides stable attachment to the amputee’s residual limb, and the knee connector integrates with a modular prosthetic knee. In conventional prostheses, the hip joint’s movement center is typically offset laterally on the prosthetic socket (Point A), resulting in a knee trajectory (O_Knee-T_, I_Knee-T_) that poorly matches the natural knee path (I_Knee_). To resolve this mismatch, we used a dual-parallelogram RCM design that repositions the prosthetic femoral axis (rod PF) to align with the anatomical hip joint center (O_Hip_), while maintaining compatibility with standard socket mounting methods.

### 2.2. Control System for the Hip Disarticulation Prosthesis

**Figure 2:**
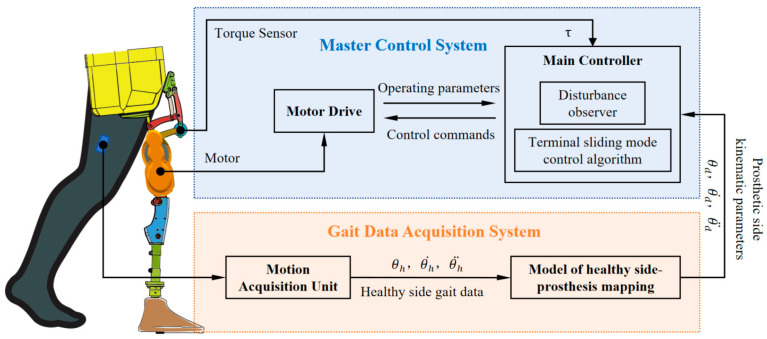
Control system block diagram of a novel powered hip disarticulation prosthesis.

Drawing inspiration from the natural symmetry of human gait—characterized by strong bilateral correlations in lower-limb kinematics—we introduce a mapping control strategy that uses the intact limb’s motion to govern the powered hip disarticulation prosthesis (Figure 2). The system integrates a posture-sensing unit for real-time acquisition of lower-limb position data, paired with a quaternion-based algorithm to extract hip joint kinematic parameters, including joint angles, angular velocities, and angular accelerations. A gated recurrent unit (GRU) neural network serves as the gait learning module, trained on bilateral hip kinematics from healthy individuals to build a kinematic mapping model between the intact and prosthetic limbs. This model uses kinematic inputs from the intact limb to predict corresponding trajectories for the prosthesis. During ambulation, real-time hip joint data from the intact side are continuously fed into the GRU model to generate motion commands for the prosthesis. A torque sensor embedded in the hip joint provides real-time feedback to the controller, enabling closed-loop regulation of prosthetic motion.

### 2.3. Kinematic Mapping Model of the Healthy Side-Prosthetic Side

#### 2.3.1. Quaternion Based Hip Joint Angle Solving

**Figure 3:**
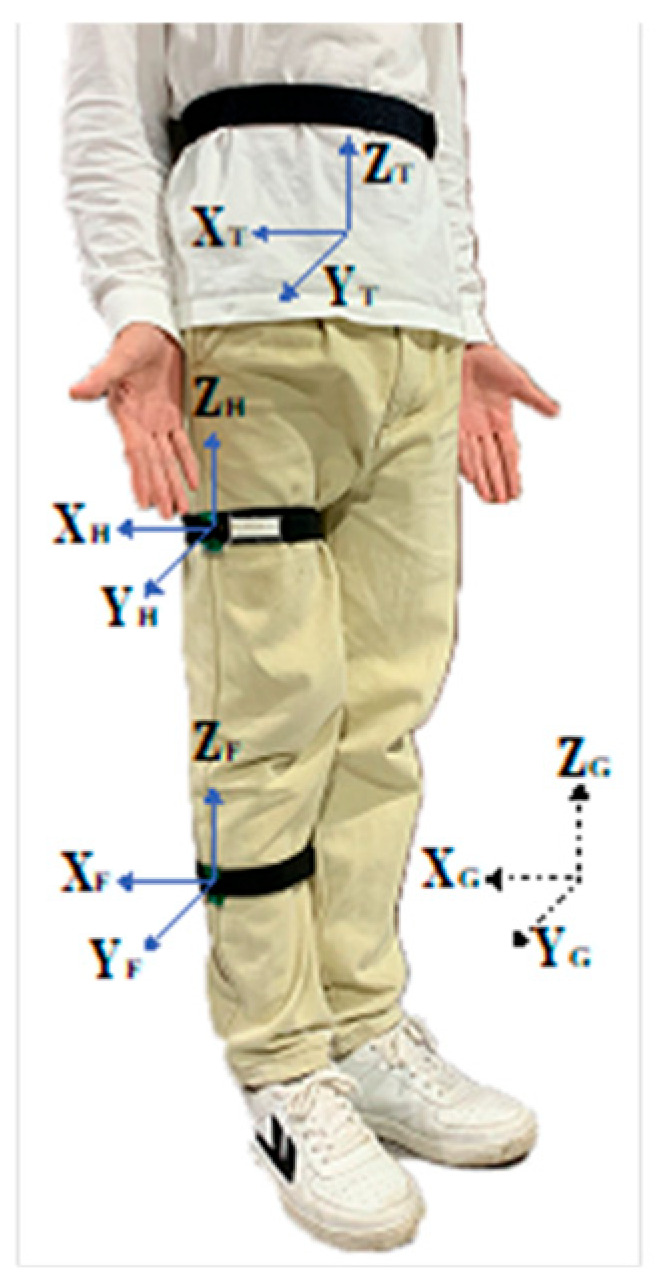
Global and local reference coordinate systems.

The posture acquisition system is illustrated in Figure 3. A global coordinate system $(X_{G},Y_{G},Z_{G})$ is established, with the Y-axis oriented laterally, the X-axis anteriorly, and the Z-axis vertically. Initially, both the femoral $(X_{H},Y_{H},Z_{H})$ and shank $(X_{F},Y_{F},Z_{F})$ local coordinate systems are assumed to coincide with the global coordinate frame. This assumption enables the construction of initial rotation matrices $R_{GT}^{i}=\left[X_{GT}^{i}Y_{GT}^{i}Z_{GT}^{i}\right]$ and $R_{GH}^{i}=\left[X_{GH}^{i}Y_{GH}^{i}Z_{GH}^{i}\right]$, where R represents the rotation matrix, the superscript “i” denotes the initial state, and subscripts “G”, “H”, and “T” refer to the global, femoral, and shank frames, respectively. The unit vectors represent the basis vectors of frame “M” expressed in frame “N”, such that $X_{MN},Y_{MN},Z_{MN}\in R^{3\times 1}$.

The inertial measurement units (IMU, model LPMS-ME1) are positioned at the waist and thigh, with coordinate systems labeled “U” and “L”, respectively. Their orientations with respect to the global frame are described by the initial rotation matrices:

(1) $$R_{UT}^{i}=(R_{GU}^{i})R_{GT}^{i},R_{LH}^{i}=(R_{GL}^{i})R_{GF}^{i}$$

When the subject moves the lower limb into a new pose, the orientations of the thigh and shank relative to the global coordinate system are given by:

(2) $$R_{GT}^{f}=(R_{GU}^{f})R_{UT}^{i},R_{GH}^{f}=(R_{GL}^{f})R_{LH}^{i}$$

where i denotes the initial moment action and f denotes the current moment action.

The posture sensor attached to the thigh provides the quaternion representation of orientation, denoted as $q={\left[x,y,z,w\right]}^{T}$, where $\left(x,y,z\right)$ forms the vector part and w is the scalar component. The quaternion can be expressed in standard form as:

(3) $$q=x_{i}+y_{j}+z_{k}+w$$

The orientation of the thigh relative to the global coordinate system can be represented by a rotation matrix derived from the quaternion $q={\left[x,y,z,w\right]}^{T}$, as follows:

(4) $$\begin{array}{cc} R_{GT}^{f} & =\left[\begin{array}{ccc} r_{11} & r_{12} & r_{13}\\ r_{21} & r_{22} & r_{23}\\ r_{31} & r_{32} & r_{33} \end{array}\right]\\ & =\left[\begin{array}{ccc} 1-2(y^{2}+z^{2}) & 2(xy-wz) & 2(wy+xz)\\ 2(xy+wz) & 1-2(x^{2}+z^{2}) & 2(yz-wx)\\ 2(xz-wy) & 2(xw+yz) & 1-2(x^{2}+y^{2}) \end{array}\right] \end{array}$$

The three Euler angles α,β,γ corresponding to yaw, pitch, and roll, respectively, describe the rotation between coordinate frames {A} and {B}. The rotation matrix can be decomposed as:

(5) $$\begin{array}{cc} R_{GT}^{f} & =R_{z}(\alpha )R_{y}(\beta )R_{x}(\gamma )\\ & =\left[\begin{array}{ccc} c\alpha c\beta & c\alpha c\beta s\gamma +c\alpha c\gamma & c\alpha s\beta c\gamma +s\alpha s\gamma \\ s\alpha c\beta & s\alpha s\beta s\gamma +c\alpha c\gamma & s\alpha s\beta c\gamma +c\alpha s\gamma \\ -s\beta & c\beta s\gamma & c\beta c\gamma \end{array}\right] \end{array}$$

where cβ denotes cosβ and sβ denotes sinβ.

The three primary hip joint angles (flexion/extension (α), abduction/adduction (β), and internal/external rotation (γ)) can be determined from the following expressions:

(6) $$\left\{\begin{array}{c} \alpha =A\tan2(r_{23}/s\beta ,r_{13}/s\beta )\\ \beta =A\tan2(\sqrt{r_{31}^{2}+r_{32}^{2}},r_{32})\\ \gamma =A\tan2(r_{32}/s\beta ,-r_{31}/s\beta ) \end{array}\right.$$

#### 2.3.2. Kinematic Mapping Model Based on GRU Network

**Figure 4:**
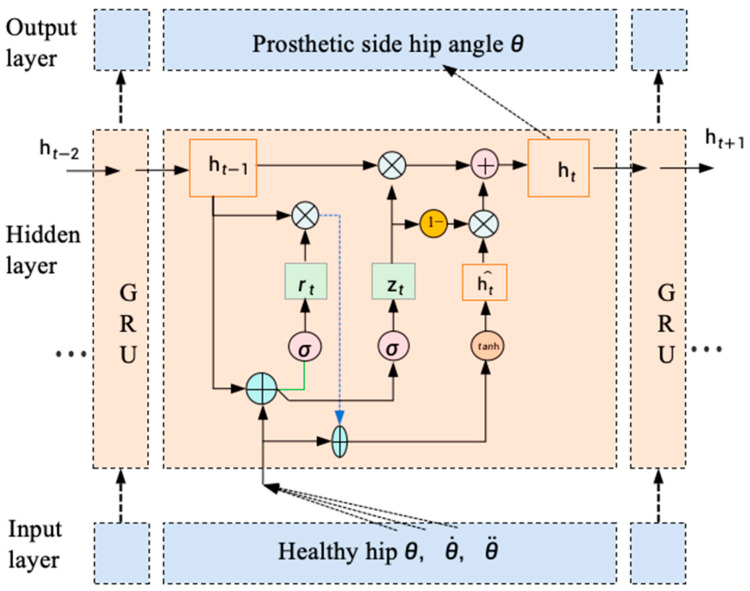
GRU network structure diagram.

During walking, hip joint angles represent the limb’s positional state, while angular velocity and acceleration characterize joint dynamics and torque behavior. In this study, all three kinematic features (joint angles, velocities, and accelerations) from both limbs were used as input to GRU neural network to construct a kinematic mapping model between the intact and prosthetic hips (Figure 4). The trained model receives intact-limb kinematic sequences and predicts corresponding motion trajectories for the powered prosthetic joint in real time.

The GRU network employs a gating mechanism to update its hidden state, allowing dynamic integration of historical context and new input at each time step. The hidden state update is computed as:

(7) $$h_{t}=(1-z_{t})⊗h_{t-1}+z_{t}⊗\hat{h_{t}}$$

where $h_{t-1}$ is the hidden state at the previous time step, ⊗ indicates the product of vectors, $\hat{h_{t}}$ is the candidate activation.

The candidate activation $\hat{h_{t}}$ is computed using the current input $x_{t}$, the previous hidden state $h_{t-1}$, and the reset gate $r_{t}$:

(8) $$\hat{h_{t}}=\tan h(W_{xc}x_{t}+W_{hc}(r_{t}⊗h_{t-1})+b_{c})$$

The update gate $z_{t}$, which controls the degree to which the hidden state is updated with new information, is defined as:

(9) $$z_{t}=\sigma (W_{xz}x_{t}+W_{hz}h_{t-1}+b_{z})$$

The reset gate $r_{t}$, determining how much of the previous state to forget, is given by:

(10) $$r_{t}=\sigma (W_{xr}x_{t}+W_{hr}h_{t-1}+b_{r})$$

where σ(*) is a Logistic function with an output interval of (0, 1), $W_{xz},W_{hz},W_{xr},W_{hr}$ are weight vectors, and $b_{z},b_{r}$ are bias vectors.

## 3. Experiments and Results

### 3.1. Participants

This study involved twelve healthy individuals and one participant with a hip disarticulation amputation. Informed consent was obtained from all participants prior to the experiments. The healthy cohort included six males and six females, with a mean age of 23.1 ± 2.8 years, average height of 171.8 ± 9.5 cm, and mean weight of 64.8 ± 9.1 kg. The amputee was a 65-year-old male (height: 175 cm, weight: 51 kg) who had undergone right hip disarticulation three decades earlier and had regularly used a 7E7-type prosthesis. The Ethical approval was obtained from the Ethics Committee of Shanghai University of Medicine & Health Sciences (Approval No. 2019-ZYXM1-04-420300197109053525), and all experiments were conducted at the Shanghai Institute of Rehabilitation Engineering.

### 3.2. Kinematic Mapping Model Prediction Experiment

**Figure 5:**
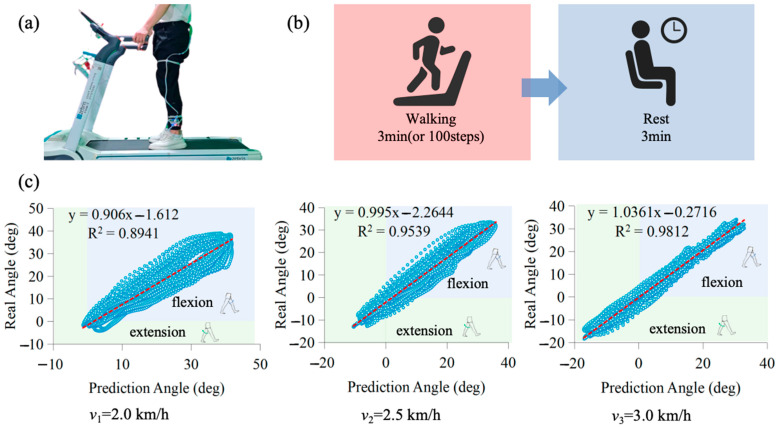
Healthy subjects experiment and results: (**a**) Subjects wearing an IMU-based posture acquisition system; (**b**) Experimental paradigm for gait data acquisition; (**c**) Mapped hip trajectories at different speeds compared with healthy human lower limb trajectories.

The twelve healthy participants wore a posture acquisition system to record bilateral gait data (including hip joint angles, angular velocities, and angular accelerations) during treadmill walking. Data were sampled at 100 Hz. Each subject walked at three speeds (2.0 km/h, 2.5 km/h, and 3.0 km/h) for 3 min (or 100 steps) per trial, with three repetitions per speed and 3 min rest intervals between trials. The resulting dataset was used to train the GRU-based kinematic mapping model. Experimental results are presented in Figure 5.

In the modeling process, the right hip joint trajectory was defined as the ground truth. Left-leg hip joint angles were used as input to predict the corresponding right-leg trajectory, and prediction performance was evaluated via linear regression. A coefficient of determination $R^{2}=1$ indicates perfect agreement. As shown in Figure 5c, the GRU model’s prediction accuracy increased with walking speed, reaching a peak similarity of 98.12% at *v*_3_ = 3.0 km/h. Notably, maximum hip flexion remained relatively stable across all speeds, whereas hip extension increased with walking speed.

### 3.3. Experiment on Amputee Wear

Human gait is inherently cyclic, involving repetitive lower-limb motion and bilateral symmetry. To evaluate the walking performance of an amputee using a powered hip disarticulation prosthesis, we employed standard gait symmetry metrics [23], including the symmetry index (SI), ratio index (RI), and relative difference index (RII), defined as:

(11) $$\begin{array}{c} SI=\frac{2(X_{R}-X_{L})}{X_{R}+X_{L}}\times 100\%\\ RI=\frac{X_{R}}{X_{L}}\\ RII=\frac{X_{R}-X_{L}}{\max(X_{R},X_{L})} \end{array}$$

Here, $X_{R}$ and $X_{L}$ represent the gait parameters of the intact and prosthetic limbs, respectively, including step length, step frequency, and maximum hip angle. where “+/−” indicates the dominant leg of the lower limb. Ideal symmetry corresponds to SI=0, RI=1 and RII=0.

**Figure 6:**
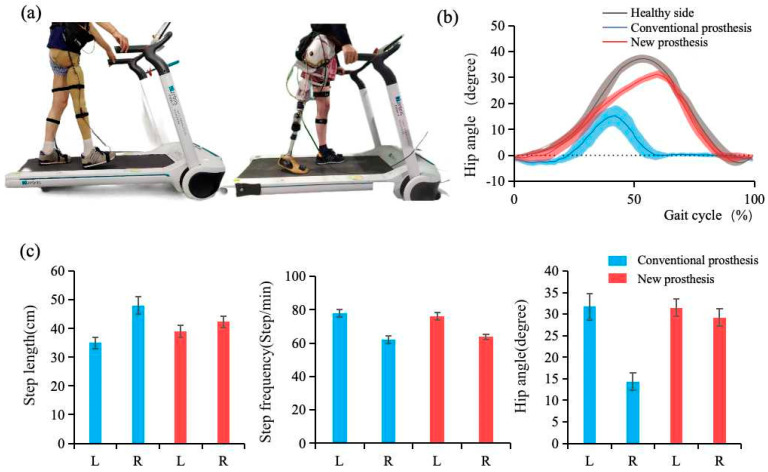
Amputee prosthetic wear test results. (**a**) Wear implementation process diagram; (**b**) Comparison of hip joint angles between two prostheses over a full gait cycle; (**c**) Gait characterization parameter mean-variance histogram.

The amputee completed walking trials using both a conventional and a powered hip disarticulation prosthesis. Hip joint kinematics were recorded using a motion capture system (Noraxon myoMOTION, Scottsdale, AZ, USA), as illustrated in Figure 6a. As shown in Figure 6b, the powered prosthesis extended the hip flexion phase from 41% to 63% of the gait cycle and increased the maximum flexion angle by 84.00% (from 17.5° to 32.2°), achieving closer alignment with the motion of the intact limb. The gait parameter variance plot in Figure 6c further highlights substantial improvements in stride length and hip angle consistency when using the powered device.

Quantitative gait analysis (Table 2) revealed notable improvements in stride length symmetry when using the powered prosthesis. Specifically, the SI decreased from 31.33% to 8.12%, and the RII from 27.08% to 7.80%, indicating a substantial reduction in bilateral asymmetry. The RI also reflects the limb dominance: for step length, RI > 1 implies the prosthetic side takes longer strides, while RI < 1 for step frequency suggests higher cadence on the healthy side. These conditions indicate compensatory mechanisms (such as heel lifting on the healthy side and pelvic elevation on the prosthetic side) to avoid toe dragging.

In terms of maximum hip angle symmetry, the conventional prosthesis exhibited a high SI of 75.65%, while the powered prosthesis reduced this value dramatically to 7.58%, reflecting a more balanced gait pattern. Furthermore, the RI improved from 0.45 to 0.93 and RII from −54.89% to −7.30%, demonstrating that the powered system more closely replicates physiological joint motion.

## 4. Discussion

### 4.1. Advantages of the Powered Prosthesis

This study presents an intelligent biomimetic gait control strategy specifically designed for hip disarticulation amputees. The approach addresses the gait abnormalities inherent to conventional prostheses by providing a stable, responsive control framework for powered hip disarticulation systems. Central to the design is an RCM mechanism, integrated with standard socket attachments, which repositions the prosthetic hip joint’s rotational center to align with the anatomical acetabulum, thereby restoring bilateral structural symmetry. Leveraging the inherent rhythmicity of human gait, we developed a kinematic mapping model that uses real-time data from the intact limb, acquired via a posture sensing system. These data are processed through a GRU network to generate control commands for the prosthetic side, achieving synchronized, biomimetic motion across both limbs.

Existing lower-limb prosthesis control methods primarily rely on physiological or mechanical signals. IMUs are widely used for pose estimation due to their stability and affordability. However, in hip disarticulation amputees, the extremely short residual limb often requires exaggerated pelvic motion to initiate stepping-compensations that deviate significantly from natural hip biomechanics. Moreover, IMU-based systems inherently suffer from latency, for which no robust mitigation strategies currently exist.

Electromyographic (EMG) signals, which can precede movement onset by 30–150 ms [24], offer another control modality and are widely used in lower-limb prosthetics. Yet EMG control faces substantial limitations, including sensitivity to electrode placement, muscle fatigue, and skin-electrode impedance [25,26]. Critically, hip disarticulation amputees often lack sufficient residual musculature to produce consistent, controllable signals.

In contrast, the proposed GRU-based kinematic mapping model leverages intact-limb motion to generate prosthetic trajectories with over 98% similarity to healthy hip kinematics. This model enables biomimetic, real-time motion control that circumvents the limitations of both mechanical and physiological signal-based systems.

Experimental results with the amputee demonstrated substantial improvements in gait symmetry when using the powered prosthesis. Stride length asymmetry was reduced from 31.33%/27.08% (conventional prosthesis) to 8.12%/7.80% (powered prosthesis), representing a reduction of 71.2% to 74.1%. Maximum hip angle deviation decreased by 89.9%, from 75.65% to 7.58%. Symmetry metrics improved markedly (from 0.45 to 0.93 (SI) and from −54.89% to −7.30% (RII)), indicating near-physiological bilateral gait. These enhancements are attributed to the active actuation mechanism and the GRU-based kinematic mapping model, which achieved 98.12% trajectory prediction accuracy. Additionally, hip flexion improved by 84.00% compared to the conventional prosthesis, effectively reducing compensatory movements. The observed increase in hip extension with walking speed is consistent with natural biomechanics, where enhanced extension contributes to forward propulsion.

Despite these advances, several asymmetries remain. The step length (RI > 1) and step frequency (RI < 1) suggest continued compensatory behaviors, such as heel lifting on the intact side and pelvic elevation on the prosthetic side, likely adopted to prevent toe drag. Moreover, an RI < 1 in maximum hip angle indicates a smaller range of motion on the prosthetic side. These residual gait deviations likely arise from insufficient actuator output, limited dynamic response, safety constraints restricting joint mobility, and suboptimal force transfer between the socket and residual limb.

### 4.2. Future Works

This study demonstrated the effectiveness of a powered hip disarticulation prosthesis and its control strategy in improving gait symmetry during level walking. However, the current applicability of the system is confined to level-ground walking and relies heavily on intact-limb motion monitoring. Its performance in multi-modal locomotion tasks—including stair ascent, descent, and traversing uneven terrain—remains unvalidated. Furthermore, this study does not address the challenge of achieving long-term adaptation and personalization for the prosthesis.

Future efforts will focus on three key areas: (1) developing AI-driven adaptive control algorithms to dynamically tune parameters and reduce compensatory behaviors; (2) enabling seamless transitions across locomotion modes to improve real-world usability; and (3) designing hybrid active/passive prostheses to overcome practical constraints related to battery life, device weight, motor size, and energy efficiency, ultimately enhancing human–robot interaction.

## 5. Conclusions

This study presents a physiologically inspired solution for hip disarticulation prostheses by integrating biomimetic mechanical design with deep learning-based control. The use of a RCM mechanism restores the hip joint’s rotational center to the anatomical acetabulum, re-establishing bilateral structural symmetry. A GRU-based kinematic mapping model, trained on intact-limb motion, achieved a trajectory prediction accuracy of 98.12%, which can meet the demand of intelligent prosthetic control. Experimental validation showed that the powered prosthesis reduced step length asymmetry by 71.2–74.1%, improved maximum hip angle symmetry by 89.9%, and increased the symmetry index from 0.45 to 0.93 substantially minimizing compensatory movements. However, there is still an asymmetry of about 7.3% in the joint range of motion, which suggests that the current system still needs to be optimized in terms of drive power, response speed, and safety strategy.

## Figures and Tables

**Table 1:** Comparison with Conventional Hip Disarticulation Prostheses.

Prosthesis Type		Features	Advantages	Limitations
Passive Prosthesis	[7,8]	Purely mechanical structure	Lightweight with excellent support	No power, No intelligent control, Difficult to operate
Intelligent Prosthesis	[9]	Powered Prosthesis	Provides leg-lifting power	Excessive weight, restricted mobility
	[10]	Powered Prosthesis	Provides leg-lifting power	Low biomimetic fidelity, limited durability

**Table 2:** Quantification of gait symmetry metrics.

Gait Parameters	Conventional Prosthesis			New Prosthesis		
	SI	RI	RII	SI	RI	RII
Step length	31.33%	1.37	27.08%	8.12%	1.08	7.80%
Step frequency	−22.62%	0.80	−20.33%	−17.65%	0.84	−16.22%
Hip trajectory	−75.65%	0.45	−54.89%	−7.58%	0.93	−7.30%

## Data Availability

The data supporting this study’s findings are available upon reasonable request from the authors.
